# Mortality in Eosinophilic Esophagitis – a nationwide, population-based matched cohort study from 2005 to 2017

**DOI:** 10.48101/ujms.v126.7688

**Published:** 2021-08-31

**Authors:** Lovisa Röjler, John J. Garber, Bjorn Roelstraete, Marjorie M. Walker, Jonas F. Ludvigsson

**Affiliations:** a Department of Pediatrics, Örebro University Hospital, Sweden; b Gastrointestinal Unit, Massachusetts General Hospital, Harvard Medical School, Boston MA, USA; c Department of Medical Epidemiology and Biostatistics, Karolinska Institutet, Stockholm, Sweden; d Department Anatomical Pathology University of Newcastle Faculty of Health and Medicine School of Medicine and Public Health Callaghan, NSW, Australia; e Division of Epidemiology and Public Health, School of Medicine, University of Nottingham, City Hospital, Nottingham, UK; f Celiac Disease Center, Department of Medicine, Columbia University College of Physicians and Surgeons, New York, New York, USA

**Keywords:** death, cancer, eosinophilic esophagitis, mortality, population-based

## Abstract

**Background:**

There is a lack of knowledge about mortality in eosinophilic esophagitis (EoE). Therefore, this study aimed to examine the mortality in EoE.

**Methods:**

A nationwide, population-based matched cohort study was conducted of all EoE patients in Sweden diagnosed between July 2005 and December 2017. Individuals with EoE (*n* = 1,625) were identified through prospectively recorded histopathology codes from all gastrointestinal pathology reports in Sweden, representing 28 pathology departments (the ESPRESSO study). Each individual with EoE was then matched with up to five reference individuals from the general population (*n* = 8,003) for age, sex, year of birth, and place of residence. We used the Cox proportional hazard modeling to estimate the adjusted hazard ratio (aHR) and 95% confidence interval (95% CI) while adjusting for other potential confounders. In sensitivity analyses, mortality in EoE patients was compared with mortality in their siblings.

**Results:**

Through December 2017, 34 deaths were confirmed in EoE patients (4.60 per 1,000 person-years) compared with 165 in reference individuals (4.57 per 1,000 person-years). This rate corresponds to an aHR of 0.97 (95% CI = 0.67–1.40). HRs were similar in males (aHR = 1.00 [0.66–1.51]) and females (aHR = 0.92 [0.38–2.18]). We observed no increased risk in mortality due to esophageal or other gastrointestinal cancers in patients with EoE (aHR = 1.02 [0.51–2.02]).

Mortality was similar in EoE patients and their siblings (aHR = 0.91 [0.44–1.85]).

**Conclusion:**

In this nationwide, population-based matched cohort study in Sweden, there was no increased risk of death in patients with EoE compared with their siblings and the general population.

## Introduction

Eosinophilic esophagitis (EoE) is a chronic inflammatory condition of the esophagus that is associated with recurrent food impaction and the development of esophageal strictures. The incidence of EoE appears to be increasing for reasons that are obscure (1). A meta-analysis of 18 studies describing the incidence of EoE in adults and children reported an overall incidence rate of 4.4 new cases of EoE per 100,000 individuals/year (2), and the disease is now estimated to affect 1 in 2,000 persons in North America and Europe (3, 4). Despite the growing recognition of EoE as an important clinical entity associated with significant disease burden and health care costs (5), the natural history of EoE is not fully known.

The most well-recognized complication of EoE is a progression from a predominantly inflammatory to a fibrostenotic phenotype (6), which is associated with the development of esophageal strictures or diffuse esophageal narrowing (7).

Straumann and colleagues described the clinical course of 30 adult EoE patients who were followed for an average of 7.2 years. The authors found no cases of esophageal malignancy or death from any cause (8). Another study followed 13 EoE patients for an average of 13.6 years. Similarly, this study did not observe any cases of malignancy or death during the 13.6-year follow-up (9). Importantly, neither the US (10) nor international (11) guidelines and consensus documents specifically address mortality in EoE. Mortality associated with EoE is presumed to be relatively low, but this has not been specifically examined in a large, geographically restricted cohort of EoE patients.

Inflammation has been hypothesized to play a role in the development of several chronic conditions associated with increased mortality, including cardiovascular disease (CVD) (12) and cancer (13). Because EoE is a life-long disease that typically afflicts young, otherwise healthy individuals, patients may experience many years of persistent and chronic immune activation and inflammation. While the direct effects of long-standing esophageal inflammation are recognized to contribute to esophageal fibrosis (6), the long-term risk of mortality associated with EoE remains elusive. We, therefore, sought to examine the mortality in 1,625 patients with biopsy-verified EoE and to compare them with 8,003 reference individuals from the general population, as well as with 2,142 siblings of the 1,625 EoE patients.

## Methods

### Study population

#### Eosinophilic esophagitis

**Figure 1:**
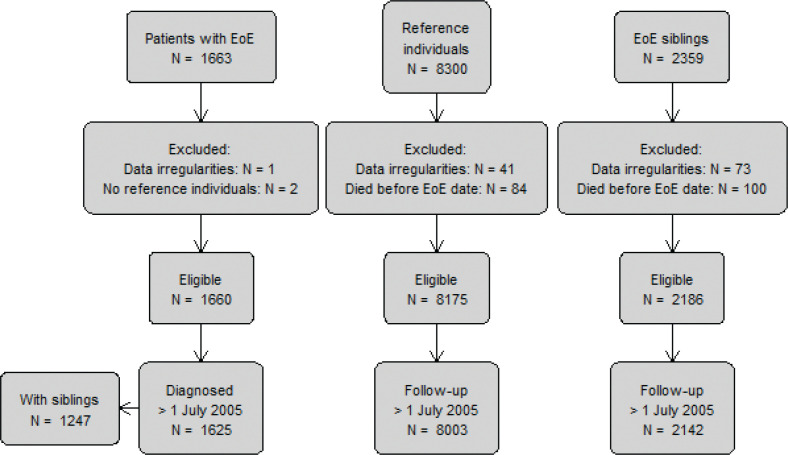
Flowchart of study participants.

Swedish biopsy data are categorized according to the SNOMED-CT classification system (Systematized Nomenclature of Medicine Clinical Terms), a standardized health care terminology used in many countries. Through the previously described ESPRESSO cohort (14), we requested all electronic gastrointestinal (GI) histopathology reports of esophageal biopsies (T62) obtained at 28 centers across Sweden from October 12, 2015 through April 10, 2017. We limited EoE to incident disease from July 1, 2005 onwards since data on medications were available only since that date. Cases of EoE (*n* = 1,625; Figure 1) were identified based on the presence of inflammation that included eosinophils (M47150). Data from our group show that the presence of eosinophilic inflammation on histopathology has a positive predictive value of 89% for the EoE diagnosis (15). For a more detailed review of our data collection, we refer to our previous publication on the ESPRESSO study (14).

#### Reference individuals

Each individual with EoE was matched with up to five age, sex, county of residence, and birth year reference individuals (*n* = 8,003) from the Swedish Total Population Register (TPR) (16). Reference individuals had to be free from EoE at the time of matching but could still be included in the study if they developed EoE in the future. However, if they developed EoE later, their follow-up was excluded from the control group.

#### Sibling comparators

We identified siblings of the EoE patients (*n* = 2,142) through the Swedish Multigeneration Register, a sub-section of the TPR. Sibling data were available on all individuals born after 1932 and who were registered as residents of Sweden in 1961 or later. To minimize intrafamilial confounding (mainly genetic and early environmental factors) that could potentially influence both the risk of EoE and mortality, sibling comparators were examined.

### Outcome measure

Date of death was retrieved from the TPR. This register is maintained by the Swedish government and contains data on life events, including birth, death, family relationships, and migration within as well as migration to and from Sweden. It covers essentially 100% of deaths (16). The Swedish Cause of Death Register (17), a comprehensive and virtually complete record of all deaths in Sweden since 1952, contains data on the cause of death. Our primary outcome was overall mortality, but we also examined death from CVD, cancer, and other diseases (including GI and infection-related deaths).

### Other covariates

Level of education was divided into the following categories: compulsory (≤9 years), upper secondary (10–12 years), and college or university (≥13 years). Data were retrieved from the Longitudinal Integrated Database for Health Insurance and Labour Market Studies (LISA) (18).

To assess the potential importance of drug treatment in EoE and mortality, we estimated separate HRs for death in EoE patients with and without steroids and proton-pump inhibitors (PPIs). Medication data were available through the Swedish Prescribed Drug Register (19), and the analysis was, therefore, restricted to incident EoE patients from July 1, 2005 or later, when the register started. Steroid use was defined as having an ATC code of H02AB (systemic), R03BA01, R03BA02, R03BA05, R03BA08, and R01AD09 (all R-codes represented swallowed/topical steroids) from first EoE diagnosis or January 1, 2006 (we allowed half a year since the start of the register to make sure this was the first ever steroid prescription) up until death. We defined PPIs similarly but through ATC code A02BC.

### Statistical analysis

The study design was a population-based, nationwide matched cohort study. In our primary analysis, EoE patients and general population reference individuals were matched (1:5) at the time of diagnosis for age, sex, county of residence, and calendar year. Study follow-up was done from the date of diagnosis of EoE (or index date for matched reference individuals) to the date of death, emigration, or end of follow-up on December 31, 2017, whichever came first. For the reference individuals, there was also the possibility that they might develop EoE during follow-up; if this occurred, they were excluded as reference individuals and moved to the EoE group. Cox proportional hazard modeling was used to calculate the hazard ratio (HR) and 95% confidence interval (95% CI) for overall and cause-specific mortality while accounting for the matching variables. Absolute risks (deaths per 1,000 person-years of follow-up) were calculated for the complete follow-up period. In exploratory analyses, the association was examined according to strata defined by years of follow-up (divided into three groups), age at first EoE diagnosis (<18, 18 to <50, and ≥50 years), sex, and education level (Table 1).

**Table 1:** Demographics for patients with eosinophilic esophagitis and reference controls (general population, normal biopsy, and siblings).

	EoE	Population reference individuals	Siblings
	*n* (%)	*n* (%)	*n* (%)
**Total**	1,625 (100.00)	8,003 (100.00)	2,142 (100.00)
Male	1,221 (75.14)	6,004 (75.02)	1,097 (51.21)
Female	404 (24.86)	1,999 (24.98)	1,045 (48.79)
**Age at start of follow-up (years)**			
Mean (SD)	37.71 (20.34)	37.35 (20.16)	37.81 (19.81)
Median (IQR)	39.00 (19.00–53.00)	38.00 (19.00–52.50)	39.00 (21.00–53.00)
<18	247 (15.20)	1,226 (15.32)	257 (12.00)
18 to <50	824 (50.71)	4,100 (51.23)	1,131 (52.80)
**≥**50	554 (34.09)	2,677 (33.45)	754 (35.20)
**Years of follow-up (years)**			
Mean (SD)	4.55 (2.43)	4.51 (2.44)	4.59 (2.35)
Median (IQR)	4.09 (2.71–6.07)	4.07 (2.64–5.93)	4.21 (2.84–5.99)
<1	32 (1.97)	210 (2.62)	49 (2.29)
1 to <5	996 (61.29)	4,886 (61.05)	1,283 (59.90)
**≥**5	597 (36.74)	2,907 (36.32)	810 (37.82)
**Start of follow-up**			
2005–2011	421 (25.91)	2,075 (25.93)	547 (25.54)
2012–2013	436 (26.83)	2,155 (26.93)	630 (29.41)
2014–2015	566 (34.83)	2,782 (34.76)	717 (33.47)
2016–2017	202 (12.43)	991 (12.38)	248 (11.58)
**Reason for end of follow-up**			
Death	34 (2.09)	165 (2.06)	35 (1.63)
Emigration	13 (0.80)	126 (1.57)	9 (0.42)
December 31, 2017	1,578 (97.11)	7,709 (96.33)	2,092 (97.67)
Diagnosed with EoE	0 (0.00)	3 (0.04)	6 (0.28)
**Education**			
Compulsory school (**≤**9 years)	254 (15.63)	1,511 (18.88)	315 (14.71)
Upper secondary school (10–12 years)	574 (35.32)	2,802 (35.01)	782 (36.51)
College or university (**≥**13 years)	488 (30.03)	2,044 (25.54)	638 (29.79)
No data	309 (19.02)	1,646 (20.57)	407 (19.00)

EoE, eosinophilic esophagitis; SD, standard deviation; IQR, interquartile range.

In the secondary analysis, the rate of mortality in EoE patients was compared with their siblings and to patients with normal esophageal biopsies. Sibling analyses were stratified as per family (one stratum per family). The power of this approach is that it automatically controls for covariates that are shared in the family (family situation, genetics, etc.). Finally, we also explored mortality in EoE according to steroid use (see Appendix for a list of relevant ATC codes). In a posthoc analysis, we also compared mortality in EoE patients with systemic steroids vs those with swallowed/topical steroids.

All analyses were adjusted for age, sex, county of residence, and year of biopsy. Statistics were carried out using R statistical software (version 3.5.2; R Foundation for Statistical Computing, Vienna, Austria) and the survival package (version 2.43, Therneau, T (2015), https://CRAN.R-project.org/package=survival). Statistical significance was set to *P* < 0.05. CIs were computed by inversion of the likelihood ratio test statistic.

### Ethics

This study was approved by the Stockholm Ethics Review Board. An informed consent was waived by the board, given that the study was strictly register based (20).

### Patient and public involvements

Patients or the public were not involved in the design, or conduct, or reporting, or dissemination plans of our research.

## Results

### Background data of EoE patients and reference individuals

In total, 1,625 patients were diagnosed with EoE over 12 years (2005–2017) (Table 1). The median age at diagnosis was 39 years (interquartile range [IQR] 19–53). Half of the patients were between 18 and 50 years old, with 15.2% of EoE patients diagnosed before the age 18 and 34% being ≥50 years at the time of diagnosis. Consistent with previous descriptions, an observed male predominance (75.1%) was noted. The median duration of follow-up was 4.09 years (IQR 2.71–6.07), with 42 (2.6%) EoE patients being followed for ≥10 years. Some 370 (22.87%) of EoE patients had a record of steroid use after EoE diagnosis. A patient chart review from the ESPRESSO cohort has revealed that in a random subset of 54 EoE patients with data on the location of esophageal biopsy, 54% had biopsies taken both in the upper/mid part and the lower part of the esophagus (15).

### Mortality among EoE patients and general population reference individuals

**Figure 2:**
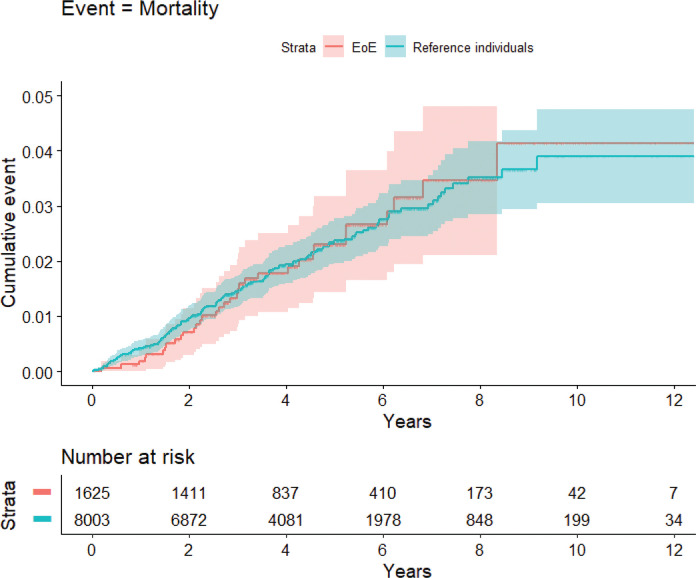
Kaplan–Meier curves for death in eosinophilic esophagitis compared with general population reference individuals.

**Figure 3:**
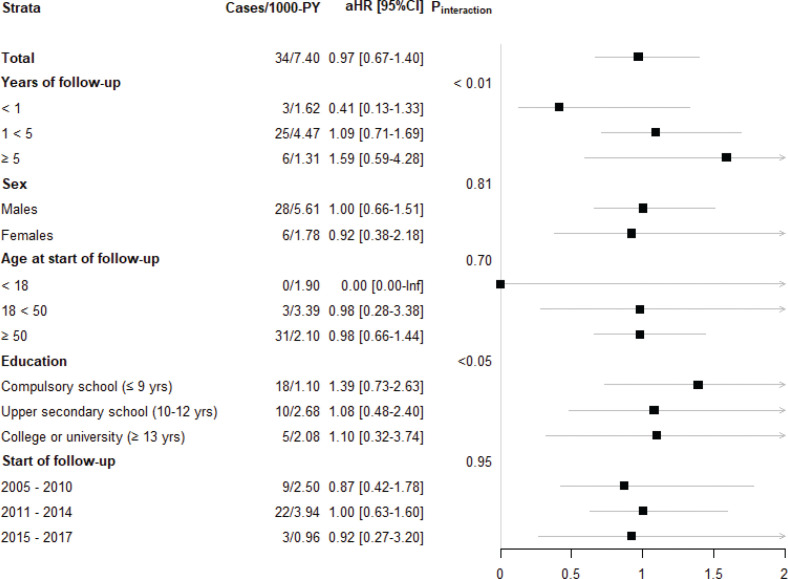
Incidence and adjusted hazard ratios for death in patients with eosinophilic esophagitis compared with general population reference individuals.

In the EoE group, 34 deaths were confirmed (4.6 per 1,000 person-years) compared with 165 in population comparators (4.57 per 1,000 person-years) (Table 2). These risk estimates correspond to an HR of 0.97 (95% CI = 0.67–1.40) (Figures 2 and 3; eTable 1 shows crude HRs and eTable 2 shows adjusted HRs). Individuals with EoE died from a wide range of causes, including cancer, CVD, pulmonary disease, and infections.

**Table 2:** Mortality incidence rates with 95% CI per 1,000 person-years for patients with eosinophilic esophagitis and reference controls (general population, normal biopsy, and siblings).

	EoE	Population reference individuals	Siblings
***N* Total**	1,625	8,003	2,142
*N* events	34	165	35
Incidence proportion (%)	2.09	2.06	1.63
Person-years	7,399	36,112	9,823
Incidence rate/1,000 person-years (95% CI)	4.60 (3.30–6.26)	4.57 (3.92–5.29)	3.56 (2.57–4.84)
**Years of follow-up**			
<1	1.85 (0.67–4.46)	4.15 (2.96–5.68)	3.75 (1.93–6.76)
1 to <5	5.60 (3.80–7.99)	5.04 (4.19–6.03)	3.32 (2.16–4.93)
≥5	4.58 (2.15–8.91)	3.46 (2.30–5.05)	4.19 (2.07–7.81)
**Sex**			
Males	4.99 (3.46–7.00)	4.75 (4.00–5.60)	3.61 (2.30–5.46)
Females	3.36 (1.58–6.54)	3.99 (2.88–5.42)	3.51 (2.20–5.37)
**Age at start of follow-up (years)**			
<18	0.00 (0.00–0.00)	0.21 (0.07–0.59)	0.46 (0.11–1.70)
18 to <50	0.88 (0.32–2.13)	0.90 (0.55–1.41)	0.83 (0.33–1.81)
≥50	14.74 (10.41–20.36)	14.73 (12.55–17.20)	10.71 (7.52–14.87)
**Education**			
Compulsory school (**≤**9 years)	16.30 (10.36–24.65)	9.40 (7.38–11.81)	8.21 (4.74–13.47)
Upper secondary school (10–12 years)	3.73 (2.05–6.37)	4.69 (3.64–5.96)	4.19 (2.55–6.56)
College or university (**≥**13 years)	2.40 (1.06–4.92)	2.38 (1.56–3.50)	1.41 (0.57–3.09)
**Start of follow-up**			
2005–2010	3.60 (1.92–6.31)	4.12 (3.13–5.34)	2.18 (1.08–4.07)
2011–2014	5.59 (3.70–8.15)	5.19 (4.27–6.26)	4.43 (2.99–6.37)
2015–2017	3.11 (1.13–7.49)	3.18 (1.94–4.98)	3.34 (1.36–7.32)
**Start of follow-up** (with max 2 years of FU)			
2005–2010	5.11 (1.86–12.30)	4.51 (2.65–7.27)	2.61 (0.81–7.28)
2011–2014	2.92 (1.29–5.99)	5.86 (4.44–7.61)	4.29 (2.36–7.33)
2015–2017	3.61 (1.31–8.69)	2.95 (1.70–4.83)	2.94 (1.07–7.07)

95% CI, 95% confidence interval; P-years, person-years; FU, follow-up.

In the group of older patients (≥50 years at diagnosis), there were 31 deaths, corresponding to an adjusted HR (aHR) of 0.98; 95% CI = 0.66–1.44. In the 18–49-year age group, there were three deaths (aHR = 0.98; 95% CI = 0.28–3.38), and in the younger age group (≤17 years), no deaths occurred (Figure 3).

Mortality rates in patients with EoE did not differ by sex. Of the males with EoE, there were 4.99 deaths (95% CI = 3.46–7.00) per 1,000 person-years versus 4.75 (95% CI = 4.00–5.60) per 1,000 person-years in male reference individuals (Table 2), which corresponds to an aHR death of 1.00 (95% CI = 0.66–1.51) (Figure 3). In females with EoE, the mortality rate was 3.36 (95% CI = 1.58–6.54) per 1,000 person-years compared with 3.99 (95% CI = 2.88–5.42) per 1,000 person-years in female reference individuals (Table 2), which corresponds to an aHR death of 0.92 (95% CI = 0.38–2.18) in EoE patients versus population reference individuals (Figure 3).

Education level did not influence risk estimates (strata according to attained education: ≤9 years: aHR = 1.39 (95% CI = 0.73–2.63); 10–12 years: aHR = 1.08 (95% CI = 0.48–2.40); and ≥13 years: aHR = 1.10 (95% CI = 0.32–3.74)). Length of follow-up was divided into three categories: <1, 1 to <5 years, and ≥5 years. There were no differences in mortality in any of these groups (<1 year aHR = 0.41 [95% CI = 0.13–1.33]; 1 to <5 years aHR = 1.09 [95% CI = 0.71–1.69]; and ≥5 years aHR = 1.59 [95% CI = 0.59–4.28]) (Figure 3).

In the most recent subset of patients (the 202 EoE patients diagnosed since January 2015), the aHR was 0.92 (95% CI = 0.27–3.20) (Figure 3). Similar aHRs were found when the follow-up was restricted to the first 2 years (to make the different calendar periods more comparable as an earlier follow-up would otherwise influence mortality much more in the later calendar period with the shortest follow-up, namely, 2015–17) (eTable 2).

### Cause-specific mortality

Rates of death were specifically analyzed for CVD and cancer (Table 3). For the EoE patients, there were six deaths (per 7,400 person-years) attributable to cardiovascular causes, which corresponds to an aHR of 0.75 (95% CI = 0.32–1.79) as compared with reference individuals. There were 10 deaths due to cancer (per 7,400 person-years) in the EoE cohort, which corresponds to an aHR of 1.06 (95% CI = 0.53–2.11) compared with reference individuals. The number of deaths attributable to infections and GI causes was too few to allow meaningful analyses, and these were, therefore, grouped with all other causes, of which there were 18 cause-specific deaths (per 7,400 person-years) with an aHR of 1.01 (95% CI = 0.60–1.68).

**Table 3:** Causes of death, number of events, number of person-years, and crude and adjusted hazard ratios.

Cause of death	Model	Reference	Population reference individuals	Siblings
**All**	HR	34/7.40	165/36.11, 1.01 (0.70–1.46)	35/9.82, 0.76 (0.38–1.51)
	aHR	34/7.40	165/36.11, 0.97 (0.67–1.40)	35/9.82, 0.91 (0.44–1.85)
**Cardiovascular**	HR	6/7.40	40/36.11, 0.73 (0.31–1.73)	9/9.82, 0.00 (0.00–inf)
	aHR	6/7.40	40/36.11, 0.75 (0.32–1.79)	9/9.82, 0.00 (0.00–inf)
**Cancer**	HR	10/7.40	45/36.11, 1.09 (0.55–2.15)	12/9.82, 0.81 (0.25–2.59)
	aHR	10/7.40	45/36.11, 1.06 (0.53–2.11)	12/9.82, 1.05 (0.31–3.61)
**Other**	HR	18/7.40	80/36.11, 1.10 (0.66–1.83)	14/9.82, 1.28 (0.51–3.22)
	aHR	18/7.40	80/36.11, 1.01 (0.60–1.68)	14/9.82, 1.81 (0.62–5.27)

HR, crude hazard ratio; aHR, adjusted hazard ratio.

### Additional analyses

Finally, we compared 1,247 patients with EoE who had ≥1 sibling (n = 2,142) (Figure 1). The mortality in the EoE patients did not differ from that in their siblings (aHR = 0.91 [95% CI = 0.44–1.85]) (eTable 2). Adjusting our sibling analyses for education yielded a similar aHR (1.16; 95% CI = 0.54–2.46).

Restricting our cohort to EoE patients with a record of steroids, we found no association with death (HR = 0.98; 95% CI = 0.43–2.22). An interaction test found a higher mortality in EoE patients with systemic steroids (HR = 2.01; 0.94–4.29) than in those with topical/swallowed steroids (HR = 0.14; 95% CI = 0.02–1.05) (*P* = 0.016), but none of the risk estimates was statistically significant compared to the general population.

The HR for death was 1.00 (95% CI = 0.66–1.51) in EoE patients without a record of steroid medication.

## Discussion

In this nationwide population-based matched cohort study of more than 1,600 patients with EoE, we found neither increased risk of death nor any increase in death from cancer or CVD. These results are reassuring for patients who often suffer from food impaction and feeding difficulties, in which ongoing inflammation, albeit local, often leads to strictures that cause mechanical obstruction. In the present study, cases of EoE were identified based on the presence of inflammation that included eosinophils.

An accurate understanding of the long-term prognosis of EoE is critical to helping providers and patients decide among current medical and dietary treatments (21), as well as to guide priorities of wider research and therapeutic development. Over the past decade, important progress has been made toward understanding the genetic and environmental basis of the disease (22). Moreover, the definition and diagnostic criteria of EoE have evolved from this work (11). The natural history of EoE has been studied (23–25), and a range of possible outcomes described, with fibrostenotic progression being the primary complication of undiagnosed or untreated disease (6). However, one important area in the natural history of EoE that remains relatively unexamined is whether this disease carries an increased risk of mortality. Mortality in EoE has been presumed to be low, but up to this point, long-term outcomes in EoE specific to mortality have only been described in a small number of EoE patients followed longitudinally (8, 9). Through the ESPRESSO study (14), we identified more than 1,600 EoE patients and had an 80% power to detect a 25% increased risk of death at a significance level of 0.05. In the present study, we found no difference in overall mortality (HR = 0.97) in EoE patients when compared with reference individuals. In addition, the mortality rate in EoE patients did not appear to be significantly different in comparison with the general population based on the age at diagnosis, duration of follow-up or socioeconomic status.

A high rate of concurrent atopic disease (60–70%) (26) occurs in EoE and EoE patients often have atopic dermatitis, asthma, or both (26). Patients with severe atopic dermatitis may be at increased risk of death (27). Of note, however, Danish researchers have shown that (cardiovascular) mortality in atopic dermatitis is neutral in mild disease. Meanwhile, the excess mortality seen for severe disease completely disappears with adjustment for background factors (28). Multiple epidemiologic studies have confirmed increased mortality with asthma (29). Although EoE and asthma sometimes coexist and are mechanistically overlapping atopic diseases, it has not yet been established where EoE fits in the spectrum of possible mortality. We have shown here that despite often severe symptoms, patients with EoE are at no increased risk of death or, at most, have a marginally increased risk (in this study, the upper 95% CI was 1.4).

Consistent with previous research (30), there appears to be no increased mortality due to cancers, including GI cancers (e.g. esophageal carcinoma). This finding is notable because one of the strongest risk factors for esophageal adenocarcinoma is Barrett’s esophagus (31), which is thought to arise primarily from molecular genetic changes induced by chronic inflammation (acid reflux, alcohol, and smoking) in the esophageal epithelium (32). A close relationship and overlap between acid reflux and EoE have been reported, including clinical presentation and response to proton pump inhibitor (PPI) therapy (33, 34). One might expect that chronic inflammation, which is due to EoE, might also predispose to metaplastic changes giving rise over time to esophageal cancers. Our data should provide clarity and reassurance to patients given that we observed no increased risk in mortality due to esophageal or other GI cancers in patients with EoE (HR = 1.02 [95% CI = 0.51–2.02]).

The major strength of our study is that it represents the first effort to examine specifically mortality rates in EoE. Second, it uses a large cohort of EoE patients identified through a validated histopathologic method from a nationwide population-based register. The cohort of 1,625 EoE patients that we identified through histopathologic criteria was highly representative of descriptions of EoE in the literature, including a 3:1 male-to-female ratio, that the disease occurs most frequently in the early and middle decades of life, and with typical rates of exposure to conventional therapies, such as swallowed steroids (38%) and PPI antacids (87%) (15). A set of reference individuals from the general population was generated and matched for age, sex, country of residence, and year of birth. In addition to comparing EoE patients with the general population, unique access to the Swedish Multigeneration Register (a part of the larger TPR) enabled the identification of 2,142 siblings of EoE patients to compare mortality. EoE is strongly driven by both genetic and environmental factors (35), which can be difficult to isolate because genetically related family members very often live in the same house and have shared dietary and environmental exposures. The use of sibling comparators in these analyses minimizes these overlapping and potentially confounding intrafamilial factors and allows better control over isolating the direct effect of having EoE on the risk of death, which was overall similar in individuals with EoE and their siblings.

EoE was identified through a computerized search of all 28 pathology departments in Sweden. Thus, we were able to minimize selection bias that is often typical of tertiary referral centers. Moreover, this study was preceded by a careful validation of the diagnosis in 111 individuals with a history of EoE. The positive predictive value (PPV) for EoE was 89% (15), and gastric reflux as a cause of eosinophilic inflammation was ruled out. An 89% PPV is similar to that found for physician-assigned diagnoses (85–95%) in the Swedish National Patient Register (36), and because biopsy is a prerequisite for the diagnosis, the sensitivity for diagnosed EoE in Sweden should be close to 100%. Additional strengths include the completeness of the TPR (16) and the Cause of Death Register (17) to ascertain the primary outcome of death. Data on both EoE and death were prospectively recorded in Swedish registers.

Some limitations of the study should be addressed. First, awareness of EoE in hospitals outside the university system has increased only recently, and because most of our cases were diagnosed after 2010, we had a limited follow-up (mean duration was 4.6 years). This follow-up is less than the only other existing studies that have examined mortality in EoE. In one study, 30 patients were followed for an average of 7.2 years (8) and in another 13 patients were followed for an average of 13.6 years (9). However, these studies only had an estimated total follow-up of roughly 216 and 177 person-years, respectively, which can be compared with a follow-up of 7,400 person-years in our study. Importantly, 42 (2.6%) individuals in our study were actually followed for over 10 years. Still, we acknowledge that our study has not only little information on long-term risk of death beyond 10 years with EoE but also limited power to examine follow-up specific HRs. Other limitations are our lack of data on disease severity and symptoms. However, we found similar mortality HRs in those with and without steroid medications and, therefore, cannot draw any firm conclusions based on risk estimates; medications may also mirror disease activity itself.

In conclusion, in the first nationwide study of more than 1,600 individuals with biopsy-verified EoE, we found no association between EoE and death. The overall low mortality associated with EoE was confirmed after adjusting for factors known to be associated with mortality risk, as well as by minimizing intrafamilial factors through analysis of a large cohort of EoE siblings.

## Data Availability

Other researchers can apply for our data through the Swedish National Board of Health and Welfare.
